# Factors influencing the cell adhesion and invasion capacity of *Mycoplasma gallisepticum*

**DOI:** 10.1186/1751-0147-55-63

**Published:** 2013-09-05

**Authors:** Ursula Fürnkranz, Karin Siebert-Gulle, Renate Rosengarten, Michael P Szostak

**Affiliations:** 1 Department of Pathobiology, Institute of Bacteriology, Mycology and Hygiene, University of Veterinary Medicine Vienna, Veterinaerplatz 1, A-1210 Vienna, Austria

**Keywords:** Extracellular matrix, Cell adhesion, Cell invasion, R_low_, R_high_

## Abstract

**Background:**

The cell invasiveness of *Mycoplasma gallisepticum,* the causative agent of respiratory disease in chickens and infectious sinusitis in turkeys, may be a substantial factor in the well-known chronicity of these diseases and in the systemic spread of infection. To date, not much is known about the host factors and mechanisms involved in promotion or obstruction of *M. gallisepticum* adherence and/or cell invasion.

In the current study, the influence of extracellular matrix (ECM) proteins such as fibronectin, collagen type IV and heparin, as well as plasminogen/plasmin, on the adhesion and cell invasion levels of *M. gallisepticum* to chicken erythrocytes and HeLa cells was investigated *in vitro*. Two strains, R_high_ and R_low,_ which differ in their adhesion and invasion capacity, were analyzed by applying a modified gentamicin invasion assay. Binding of selected ECM molecules to *M. gallisepticum* was proven by Western blot analysis.

**Results:**

Collagen type IV, fibronectin, and plasminogen exerted positive effects on adhesion and cell invasion of *M. gallisepticum,* with varying degrees, depending on the strain used. Especially strain R_high_, with its highly reduced cell adhesion and invasion capabilities seemed to profit from the addition of plasminogen. Western and dot blot analyses showed that R_high_ as well as R_low_ are able to adsorb horse fibronectin and plasminogen present in the growth medium. Depletion of HeLa cell membranes from cholesterol resulted in increased adhesion, but decreased cell invasion.

**Conclusion:**

ECM molecules seem to play a supportive role in the adhesion/cell invasion process of *M. gallisepticum.* Cholesterol depletion known to affect lipid rafts on the host cell surface had contrary effects on cell adherence and cell invasion of *M. gallisepticum.*

## Background

For a long period of time, *Mycoplasma* spp., very small, wall-less prokaryotes, were considered obligate extracellular bacteria until in 1989 Lo *et al.* described intracellular organisms in an AIDS-patient, which were later identified as *M. fermentans*[1]. To date, other *Mycoplasma* spp., such as *M. penetrans*[2], *M. genitalium*[3], *M. pneumoniae*[4], *M. suis*[5], *M. synoviae*[6], *M. bovis*[7], *M. hyorhinis*[8], and *M. hominis*[9] have likewise been shown to be capable of invading non-phagocytic eukaryotic cells. *Mycoplasma gallisepticum*, the causative agent of chronic respiratory disease in chickens and infectious sinusitis in turkeys has been shown to be able to invade HeLa cells and chicken embryo fibroblasts (CEF) *in vitro*[10] and chicken red blood cells (RBC) *in vitro* and *in vivo*[11]. Furthermore, *M. gallisepticum* has been proven to cross the mucosal barrier and to spread systemically *in vivo*[12]. However, even closely related *M. gallisepticum* strains can differ markedly in their pathogenicity for chickens [13,14]. The strains R_low_ and R_high_ derive from different passages of strain R grown in artificial medium [14,15]. The low-passage, hemadsorption-positive and virulent strain R_low_ (10th passage) was shown to be cell-invasive *in vitro* and *in vivo*, whereas the high-passage, hemadsorption-negative strain R_high_ (164th passage) displays a highly reduced virulence and exhibits only marginal cell invasiveness [10-12,16].

Many publications over the past years addressed the bacterial proteins involved in mycoplasma cell adhesion and invasion ([17-19] for reviews). However, host factors involved in cell invasion have been investigated to a much lesser extent. The extracellular matrix (ECM) occupies the space between animal cells and is composed of secreted proteoglycans and non-proteoglycan polysaccharides or proteins like collagens, fibronectin, and laminin [20,21]. These macromolecules influence adhesion, migration, proliferation, and differentiation of eukaryotic cells [22], and they serve also as substrate for the attachment and internalization of pathogenic microorganisms [23,24]. ECM- and plasminogen-binding properties have also been reported for a limited number of *Mycoplasma* species like *M. penetrans*[25], *M. hyopneumoniae*[26,27], *M. bovis*[28] and *M. gallisepticum*[29-31]. So far, only for *M. fermentans* and *M. pneumoniae* an influence of ECM molecules and plasminogen on adhesion and invasion capabilities has been documented [32-34]. For *M. gallisepticum* strains R_low_ and R_high_, published data is limited to differential binding properties of the organism to fibronectin [29], heparin [30] and plasminogen [31].

Another host factor that plays a role in bacterial invasion processes is cholesterol, the major component of lipid rafts [35]. Cholesterol seems to play a major role in the invasion process of *M. fermentans,* as invasion rates were 70% lower in cholesterol-depleted HeLa cells, whereas adhesion rates were not influenced [32].

In the current study, our aim was to investigate hemadsorption-positive and -negative strains of *M. gallisepticum* for their capability to adhere to and invade HeLa cells and chicken red blood cells in the presence of selected ECM molecules and plasminogen. The role of cholesterol availability on the host cell membrane for the adhesion and invasion of *M. gallisepticum* was also examined.

## Materials and methods

### Cultivation of host cells and bacteria

*Escherichia coli* DH10B, *Streptococcus pneumoniae* type strain DSM20566 (DSMZ, Braunschweig, Germany) and *Streptococcus canis* strains G1 and G2 (obtained from G.S. Chhatwal, Helmholtz Center for Infection Research, Braunschweig, Germany) were used as controls in fibronectin and plasminogen binding assays. *M. gallisepticum* strains R_low_ and R_high_ were originally provided by S. Levisohn, Kimron Veterinary Institute, Bet Dagan, Israel. Mycoplasma cultures were grown in modified Hayflick medium [36] containing 20% (vol/vol) heat-inactivated horse serum (Gibco BRL, Life Technologies GmbH, Eggenstein, Germany) and 100 IU penicillin per ml (HFLX). Solid medium agar plates were produced by adding 1% (wt/vol) bacteriological agar (Agar No. 1; Oxoid Deutschland GmbH, Wesel, Germany) to HFLX. Numbers of viable bacteria [colony forming units (CFU)] were determined as described elsewhere [16].

Chicken red blood cells (RBC) from female Lohmann Brown chicken, kindly provided by C. Hess (Clinic for Avian, Reptile, and Fish Medicine, University of Veterinary Medicine Vienna, Austria), were washed twice with PBS and working suspensions were adjusted to 2 × 10^8^ RBC per ml in Dulbecco’s Modified Eagle’s Medium (DMEM, Gibco) containing 10% (vol/vol) fetal calf serum (FCS, Gibco BRL) and 5% (vol/vol) tryptose phosphate broth (Sigma). Since HeLa cells were previously used as model organisms in many mycoplasma invasion assays (e.g. [10,33,34]), we included this cell line. For the Gentamicin Invasion Assay (GIA), cells from the human epithelial-like cell line HeLa-229 (ATCC CCL-2.1; Manassas, VA) were washed three times with PBS, trypsinized for 10 min (0.05% trypsin-EDTA; Gibco BRL), subsequently seeded in 24-well cell culture plates (Greiner Bio-One GmbH, Kremsmünster, Austria; 5 × 10^4^ cells per well) and cultured for 2 days at 37°C in a 5% CO_2_ atmosphere.

### Adherence and invasion assays

The high degree of sequence homology between human, bovine and chicken fibronectin renders them experimentally interchangeable [29], which prompted us to use human fibronectin for adherence and cell invasion assays. All ECM molecules, cellular fibronectin (F2518), plasma fibronectin (F2006), collagen type IV (C5533), collagen type V (C3567) and porcine heparin (H3149), as well as plasminogen (P7999), urokinase plasminogen activator (No.124; American Diagnostica Inc., Stamford, CT), ϵ-aminocaproic acid (EACA) (A2504), and methyl-β-cyclodextrin (MßCD; C4555) were of human origin and were purchased from Sigma-Aldrich, unless otherwise indicated. The molecules were used at the following concentrations: 25 μg/ml for fibronectins, plasminogen, and collagens, 200 μg/ml for heparin, 100 μg/ml for EACA, 20 U/ml for urokinase plasminogen activator and 6 mg/ml for MβCD.

Adherence and invasion rates of *M. gallisepticum* were analyzed together in a modified GIA as described elsewhere [10,11]. Briefly, for the GIA using RBC, an overnight culture of *M. gallisepticum* was centrifuged at 10,000 × g for 10 min, resuspended in DMEM and diluted to reach a final concentration of 4 to 8 × 10^4^ CFU per ml. One ml of this suspension was mixed with 20 μl of a stock solution of the respective ECM molecules. A 250-μl aliquot of this mixture was incubated with 250 μl DMEM containing gentamicin (final concentration 400 μg/ml) for 3 h at 37°C to verify the lethal concentration of gentamicin. The residual 750 μl of the mixture was co-incubated for 2 h at 37°C with 750 μl RBC in DMEM containing 1.5 × 10^8^ RBC. A 500-μl aliquot of this mixture was then diluted 1:10 in PBS and 100-μl samples were plated on HFLX agar plates to count the number of input bacteria. The remaining ECM-mycoplasma-RBC mixture was divided into 2 samples, and centrifuged at 300 × *g* for 3 min. The resulting pellets (two per ECM molecule) were washed once with PBS to remove non-adherent bacteria, and resuspended in 500 μl DMEM each. One resuspended pellet was further diluted 1:10 in PBS and plated on HFLX agar plates to determine the total number of adherent and intracellular bacteria. The other resuspended pellet was treated with 500 μl DMEM containing gentamicin (final concentration 400 μg/ml) and incubated for 3 h at 37°C. After the gentamicin treatment, 100 μl-samples were plated to count the intracellular bacteria.

The GIA using HeLa cells was carried out in 24-well cell culture plates. If HeLa cell membranes had to be depleted from cholesterol, the cells were preincubated in the presence of 5 mM MβCD for 30 min at 37°C and 5% CO_2_ as described elsewhere [37]. After thoroughly washing to remove any MβCD, HeLa cells were co-incubated with 500 μl-samples of ECM-treated bacteria (4 to 8 × 10^4^ CFU per ml DMEM) for 2 h at 37°C in a 5% CO_2_ atmosphere (2 wells per ECM molecule). After 2 h of incubation, the HeLa cells were washed with PBS to remove non-adherent mycoplasma, trypsinized for 10 min (0.05% trypsin-EDTA; Gibco BRL) and the cells of one duplicate well were treated with 1 ml DMEM containing gentamicin (400 μg/ml) to kill all extracellular mycoplasma. After 3 h of incubation, the number of intracellular mycoplasma was determined by plating 100 μl of serial dilutions in PBS onto HFLX agar plates. The other duplicate well was used to determine the number of adherent mycoplasma and treated the same way except that no gentamicin was added. The number of input bacteria was determined from serial dilutions of mycoplasma samples before ECM treatment. All experiments were arranged in duplicate assays and repeated two (R_high_), or three (R_low_) times, respectively.

### Western blots

Lysates of whole cell protein were produced from 1-ml samples of *M. gallisepticum* grown in HFLX or *E. coli* grown in Luria-Bertani (LB) medium according standard methods. For *S. pneumoniae,* biomass was collected directly from Columbia III agar plates with 5% sheep blood (Becton Dickinson, Heidelberg, Germany). Denatured whole cell proteins were separated by SDS-gel electrophoresis and transferred to nitrocellulose membranes (Whatman, Dassel, Germany). The blots were blocked with 2% blocking reagent (Blotting Grade Blocker, Bio-Rad) and incubated with either anti-fibronectin or anti-plasminogen antibodies (ABIN125555 or ABIN285631; antibodies-online GmbH, Aachen, Germany), diluted 1:1,000 in TBS. Horseradish peroxidase-labeled goat anti-rabbit IgG (P0217; DAKO Hamburg, Germany), diluted 1:2,000, was used as the secondary antibody, and the blots were developed using 4-chloro-1-naphthol (4CN) according to the manufacturer’s instructions (Bio-Rad).

### Dot blots

One-ml samples of overnight cultures of R_high_, R_low_, *E. coli*, *S. pneumoniae* and *S. canis* were washed in equal amounts of PBS and five μl were dropped onto nitrocellulose and allowed to dry. Blocking and further processing was performed as described for Western blots.

### Statistical analysis

Invasion and adhesion frequencies are expressed as the mean ± standard deviation of *n* independent values. Statistical analysis of the data was performed running the SPSS 18.0 software package (SPSS Inc. Chicago, USA). Group means were compared by Single-factor ANOVA and *post hoc* Tukey test, and P-values of < 0.05 were considered statistically significant.

## Results

**Figure 1:**
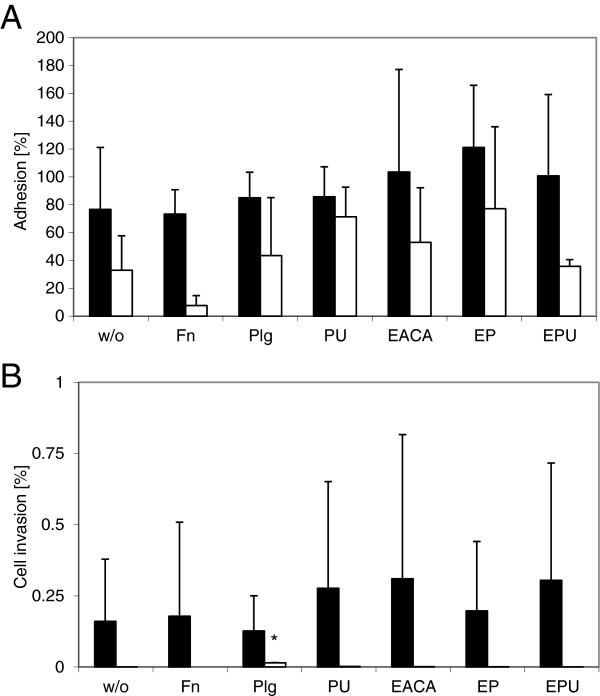
**Influence of ECM molecules on adhesion and cell invasion of *M. gallisepticum *incubated with RBC. ***Mycoplasma gallisepticum* R_low_ (solid bars) and R_high_ (open bars) were incubated with chicken RBC alone (w/o) or in combination with the ECM molecules fibronectin (Fn), plasminogen (Plg), plasminogen and urokinase plasminogen activator resulting in plasmin (PU), ϵ-aminocaproic acid (EACA), ϵ-aminocaproic acid and plasminogen (EP), and ϵ-aminocaproic acid, plasminogen, and urokinase plasminogen activator (EPU). The values for the adhesion **(A)** or cell invasion rates **(B)** represent the means of at least three independent experiments performed in triplicate (R_low_) or duplicate (R_high_) ± standard deviations. A significant difference to RBC alone (p < 0.05) is indicated by an asterisk (*).

The influence of ECM molecules and other factors on adhesion and invasion rates of two *M. gallisepticum* strains, R_low_ and R_high_, was investigated by a modified version of the Gentamicin Invasion Assay (GIA). The adhesion rate of strain R_low_ to chicken RBC was about 77%. A slight increase was seen with EACA, alone or in combination with plasminogen or plasminogen activated to plasmin by the addition of the human urokinase plasminogen activator (Figure 1A). A more remarkable increase was observed when the hemadsorption-negative and avirulent strain R_high_ was incubated together with plasmin. EACA in combination with plasminogen resulted in adhesion rates three times higher than those of R_high_ alone, and fibronectin apparently decreased the adhesion capacity of R_high_ for RBC.

However, standard deviations were high and thus differences were not statistically significant. The previously reported low cell invasion rates of R_low_ for RBC [11] could be confirmed and were not notably influenced by any ECM molecule (Figure 1B). A slight decrease in the mean invasion rate was observed in the presence of plasminogen. R_high_ showed almost no cell invasion capabilities in this set-up; only with plasminogen a significantly increased cell invasion (p < 0.05) was observed (0.014 ± 0.003%).

**Figure 2:**
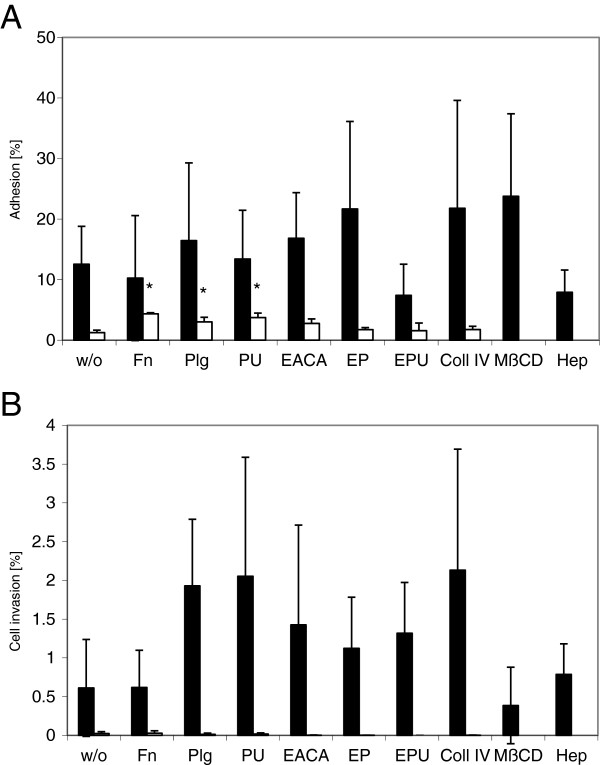
**Influence of ECM molecules on adhesion and cell invasion of *M. gallisepticum *incubated with HeLa cells. ***Mycoplasma gallisepticum* R_low_ (solid bars) and R_high_ (open bars) were incubated with HeLa cells alone (w/o) or in combination with the ECM molecules fibronectin (Fn), plasminogen (Plg), plasminogen and urokinase plasminogen activator resulting in plasmin (PU), ϵ-aminocaproic acid (EACA), ϵ-aminocaproic acid and plasminogen (EP), ϵ-aminocaproic acid, plasminogen, and urokinase plasminogen activator (EPU), collagen type IV (Coll IV) or heparin (Hep). Furthermore, HeLa cells were pre-treated with methyl-β-cyclodextrin (MβCD). The values for adhesion **(A)** or cell invasion rates **(B)** represent the means of at least three independent experiments performed in triplicate (R_low_) or duplicate (R_high_) ± standard deviations. Significant differences (p < 0.05) are indicated by asterisks.

A positive influence on the adhesion of strain R_low_ to HeLa cells was observed upon the addition of plasminogen, type IV collagen, plasminogen with EACA, and plasmin (Figure 2A). However, none of these elevations was statistically significant. R_high_ exhibited eight-fold lower adhesion rates to HeLa cells than R_low_, however, there was a statistically significant enhancement in adhesion of R_high_ when adding fibronectin (p ≤ 0.001), plasminogen (p = 0.022) or plasmin (p ≤ 0.001) in comparison to R_high_ w/o (Figure 2A). As all results obtained with cellular- or plasma-derived fibronectin were comparable, only the results for cellular fibronectin are shown in the figures.

Cell invasion rates of R_low_ into HeLa cells were increased by the addition of plasminogen, plasmin and collagen type IV (Figure 2B). The combination of EACA with either plasminogen or plasmin decreased invasion rates. Surprisingly, we also recorded enhanced cell invasion rates of R_low_ with EACA alone, compared to the mean invasion rates of R_low_ alone (Figure 2B).

To get an insight into the role of cholesterol/lipid rafts concerning the cell invasion and/or adhesion process, HeLa cell membranes were depleted from cholesterol using MβCD prior to the addition of the bacteria. Whereas cholesterol depletion had a positive effect on the adhesion of R_low_ (Figure 2A), cell invasion was slightly decreased (Figure 2B). However, the observed differences in cell invasion were not statistically significant.

**Figure 3:**
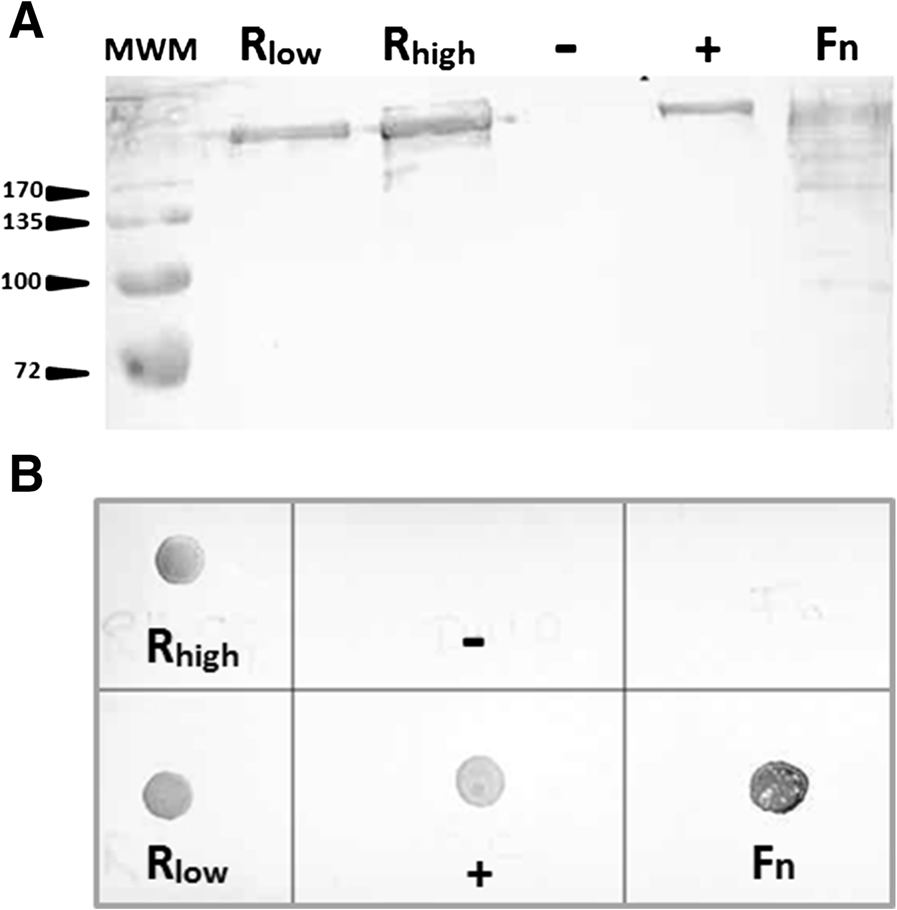
**Detection of fibronectin in cultures of R**_**low **_**and R**_**high**_**.** The occurrence of fibronectin in whole washed cells and cell lysates of *M. gallisepticum* strains R_low_ and R_high_ was analyzed in Western blot **(A)** and dot blot **(B)** assays by use of a fibronectin-specific antibody. Purified fibronectin (Fn) as well as the reference culture *S. pneumoniae* DSM20566 (+) were used as positive controls, while *E. coli* DH10B (−) served as a negative control. A high molecular weight band comigrating with purified fibronectin could be detected in cell lysates of R_low_ and R_high_**(A)**. Sizes of the molecular weight markers (MWM) are given in kDa next to the arrows. Dot blot analyses **(B)** indicated the binding of fibronectin to the bacterial cell surface of both *M. gallisepticum* strains, R_low_ and R_high_.

**Figure 4:**
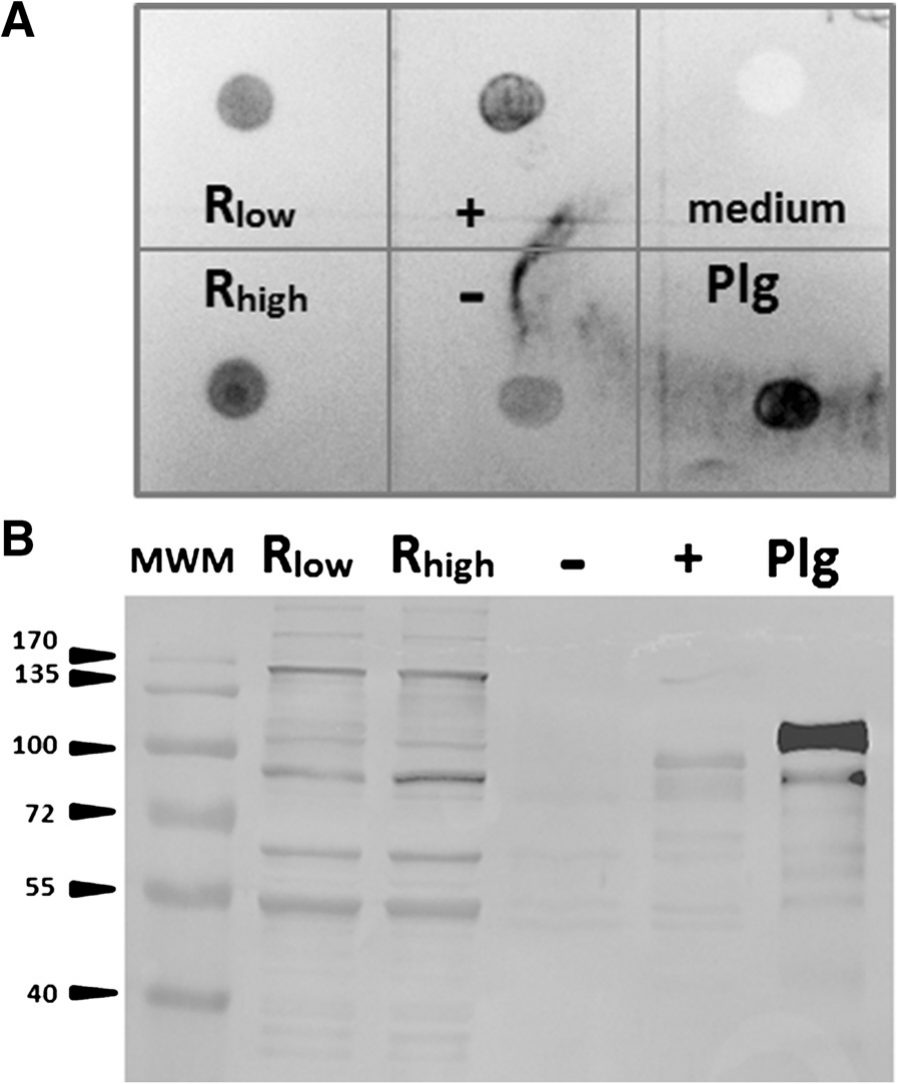
**Detection of plasminogen in cultures of R**_**low **_**and R**_**high**_**.** R_low_ and R_high_ were also equally well stained on dot blot assays using a plasminogen-specific antibody **(A)**. As positive controls *Streptococcus canis* strain G1 (+) [38] and plasminogen (Plg) were used, while *S. canis* strain G2 (−) [38] or HFLX medium served as negative controls. On Western blots, prominent protein bands of 158, 89, 64 and 53 kDa were stained equally well in cell lysates of R_low_ and R_high_**(B)**. MWM, molecular weight marker.

As the addition of fibronectin led to an increased adhesion of R_high_ to HeLa cells, while R_high_ has been reported to lack the fibronectin-binding proteins found in R_low_[29], Western and dot blot analyses of cell lysates or intact cells were performed. Fibronectin was detected on whole cells of R_low_ as well as R_high_ without the necessity of preincubating the mycoplasma cultures with supplemented fibronectin (Figure 3B). *Streptococcus pneumoniae*, a species known to possess fibronectin-binding proteins, also appeared positive on dot blots while *E. coli* proved negative. Western blot analysis of R_low_ and R_high_ cell lysates with fibronectin-specific antibodies resulted in the detection of a band at the same size as the fibronectin used as positive control or in the cell lysate of *S. pneumoniae* (Figure 3A). No difference regarding the occurrence of fibronectin in samples of R_low_ and R_high_ could be observed. When tested for the presence of plasminogen on the bacterial cell surface, both R_low_ and R_high_ proved positive in dot blot assays (Figure 4A). While *Streptococcus canis* strains G1 and G2, which were used as positive and negative controls for plasminogen binding according to a recently published report by Fulde *et al.*[38], exhibited either intense or marginal reaction with the Plg-specific antibodies, R_low_ and R_high_ showed an equally strong reaction. On Western blots, protein bands of 158, 89, 64 and 53 kDa were labeled in the cell lysates of R_low_ and R_high_ (Figure 4B).

## Discussion

ECM molecules, like fibronectin or vitronectin, have been described to play a strategic role for pathogenic bacteria. They serve as a bridge component between the pathogen that binds to the ECM molecule and to host cells, which naturally have receptors for these ECM molecules [39,40], thereby bringing the bacterium in close contact with the host cell. A more sophisticated exploit of interactions between ECM molecules has been described for *Streptococcus pyogenes* which is able to adhere to collagen via fibronectin bound to the bacterial surface [41]. Plasminogen might act similarly as a bridging component in rare events [42]. However, for cell invasive bacteria plasminogen might be of better use in its activated form, the serine protease plasmin. Besides exhibiting a crucial role in fibrinolysis and ECM remodeling, the proteolytic activity of plasmin could empower the plasmin-equipped bacterium more directly to penetrate host cells or transmigrate through monolayers, thereby assisting to cross the epithelial barrier [42-44]. *M*. *fermentans* has been shown to profit from binding to plasminogen or ECM molecules. When co-incubated with HeLa cells, *M. fermentans* exhibited an increased adherence to HeLa cells when pretreated with collagen type III or IV or plasminogen [32,34]. In this study, we report the likewise positive effect of plasminogen, as well as a slight enhancement due to fibronectin on the adherence capacity of *M. gallisepticum.* Fibronectin and plasminogen can of course be found in the serum used in the culture medium, which was proven by the ability to detect these molecules on the surface of R_low_ and R_high_ without further adding them (Figures 3 and 4). Nevertheless, to study their distinct effects on the invasion and adhesion capabilities of R_low_ and R_high_, fibronectin and plasminogen were added in abundance. The hemadsorption-positive strain R_low_ showed an increased adherence to HeLa cells when preincubated with plasminogen. Strain R_high,_ lacking the major cytadherence proteins GapA and CrmA, exhibits drastically decreased cytadherence capacities that have been well documented [45]. However, it seems that the rather marginal adhesion rates of R_high_ to HeLa cells can be enhanced by plasminogen, plasmin and fibronectin (Figure 2A). The recruitment of plasmin or fibronectin as a bridging molecule could represent a secondary anchoring system of *M. gallisepticum* besides the primary and accessory cytadhesion proteins like GapA and CrmA. As, on the other hand, adhesion rates of R_high_ with plasmin or fibronectin were still lower than adhesion rates of R_low_ alone, it seems that the loss of GapA and CrmA on bacterial site cannot be overcome by the ECM-mediated adhesion.

The positive effect of fibronectin on the adhesion capacity of R_high_ to HeLa cells was surprising, as in 2006 R_high_ was reported to lack fibronectin-binding proteins [29]. When we tested whole cells and whole cell-lysates of *M. gallisepticum* strains R_low_ and R_high_ for fibronectin-binding properties, it turned out that fibronectin could be detected on the surface of mycoplasma whole cells as well as in cell lysates, irrespective of the strain tested. In contrast to May *et al.*[29], who investigated the TX-114 insoluble proteins of *M. gallisepticum* R_high_, we did not exclude the cytoplasmic proteins from our analyses. In *M. pneumoniae,* also cytoplasmic proteins like EF-Tu and PDHB were shown to appear at the surface of the mycoplasma cell and to bind fibronectin [46]. Also in other *Mycoplasma* species like *M. genitalium,* EF-Tu was found to be present at the cell surface [47]. Interestingly, although the EF-Tu proteins of *M. genitalium* and *M. pneumoniae* share a 96% identity, the *M. genitalium* protein does not bind fibronectin. Recently, Balasubramanian *et al.* showed that 3 amino acid residues (S343, P345, and T357) of the *M. pneumoniae* EF-Tu were essential for its binding activity to fibronectin, and that these critical amino acids are changed to alanine residues in the EF-Tu of *M. genitalium*[47]. The EF-Tu of *M. gallisepticum*, on the other hand, shares a lower overall identity with the EF-Tu of *M. pneumoniae,* but is higher conserved for these three Fn-binding residues (Q343,K345, T357), leaving it open to speculate about an involvement of *M. gallisepticum* R_high_ EF-Tu in the adhesion process by fibronectin binding.

Detection of fibronectin on the surface of *M. gallisepticum,* routinely cultured in HFLX, indicates that the fibronectin concentration in the horse serum is high enough to allow the pathogen to adsorb substantial amounts. Absorbance of protein components from the growth medium by *M. gallisepticum* has already been reported for transferrin or immunoglobulins [48] and for the latter IgG-binding proteins have been recognized [49]. Whether binding of fibronectin plays a role for *M. gallisepticum in vivo*, remains to be elucidated.

The influence of another family of ECM molecules, collagen type IV and V, onto *M. gallisepticum* adhesion and invasion was also investigated in the present study. Whereas collagen type V revealed no marked effects (data not shown), type IV collagen had a slightly positive effect on the adherence and invasion capacity of R_low_ to HeLa cells. For *M. fermentans* a supporting effect of collagen type III and V on the adherence capacity to HeLa cells had been reported [34]. In contrast, collagen type IV had no effect on *M. fermentans* adherence. This difference in the mode of action concerning the same family of compounds (collagens) could perhaps be explained by the different nature of the three collagens investigated, as collagen type III and V exhibit fibrillar structures, and are mostly found in extensible connective tissue, whereas collagen type IV is rather linked to the basal lamina. Concomitantly, this difference might also reflect the different natural habitats of *M. fermentans* and *M. gallisepticum*.

Plasminogen is not an ECM molecule *per se* but after its full activation to plasmin, it exerts a serine protease function on the fibrinolytic system, as well as on many ECM molecules. Binding of plasmin or plasminogen to fibrin and other ECM molecules predominantly occurs via its high- and low affinity lysine-binding sites (LBS) [50], and therefore can be easily blocked by EACA, a lysine analogue. If plasminogen is used as a bridging molecule by *M. gallisepticum*, then binding of EACA to the LBS of plasminogen should compensate for any positive effect of plasminogen on adherence and/or invasion. The results from the invasion assays support this hypothesis, as R_low,_ pretreated with plasmin or plasminogen (Figure 2B), showed higher invasion rates to HeLa cells than the untreated control and addition of EACA indeed had a negative impact on the plasminogen-elevated invasion rates. For *M. fermentans*, plasminogen was shown to enhance cell invasion as well as cell adhesion [32,51], whereas no effect was seen with *M. pneumoniae*[33]. When we analyzed the cell adherence of *M. gallisepticum,* an increase after treatment with plasminogen or plasmin was observed (Figure 2A). To our surprise, however, EACA had no negative effect on the plasminogen-elevated cell adhesion, but rather boosted the cell adhesion. Until recently, EACA has been described as a potent inhibitor of plasminogen-binding to bacteria, [51,52], no one has yet investigated the effect of EACA alone on bacterial cell invasion or adhesion capacity. The increase in cell adhesion of R_low_ and R_high_ in the presence of EACA alone (Figure 2A) may indicate that another *M. gallisepticum* surface protein is participating in the multifactorial adhesion process. It is well established that *M. gallisepticum* contains several cell surface proteins with hemagglutinating (pMGA/VlhA [53]), or adhesive properties like Mgc2 [54], α-enolase [31] or the uncharacterized proteins P30, P48, P50, P80 [55].

Cholesterol depletion from HeLa cell membranes resulted in a decreased invasion capacity of *M. gallisepticum,* suggesting that cholesterol-rich regions in the cell membrane like lipid rafts are needed for the invasion process. Many bacteria have been described to enter host cells via receptors being present in lipid rafts and/or caveolae [reviewed by [56,57]. An alternative strategy for mycoplasma entry could be the engulfment via the lipid rafts. So far, entry into eukaryotic cells via engulfment without the use of receptors has only been described for very small objects like viruses and bacterial toxins [35]. Mycoplasmas belong to the smallest bacteria, they do not contain a cell wall, and their bodies seem easily deformable. *M. gallisepticum* has been observed to squeeze itself through 220-nm [58] and apparently even 100-nm pore filters (own observation). As lipid rafts *in vivo* have diameters up to 700 nm [57], *M. gallisepticum* are flexible and small enough to maybe enter the eukaryotic cell by squeezing through lipid rafts. As envisioned by others before [17,59] the fusogenic properties of the mycoplasma lipidic cell wall might additionally enable the pathogen to interact with the lipid-rich raft domains. While cholesterol depletion lowered the cell invasion rates of *M. gallisepticum,* adherence capabilities to HeLa cells were improved. This could be explained by the fact, that cholesterol removal from membranes results in dispersion of the raft-associated lipids and proteins [56], thus destroying the possible entry site, but at the same time generating more sites for the mycoplasma to adhere to. With *M. fermentans,* plasminogen-preincubated bacteria adhered to cholesterol-depleted HeLa cells as well as to cholesterol-containing cells, but internalization of *M. fermentans* was almost completely inhibited after cholesterol depletion [32].

## Conclusion

Taking these results together, it seems that *M. gallisepticum* is utilizing a second anchoring system besides its own primary and accessory cytadhesion proteins like GapA and CrmA that relies on the recruitment of ECM- or ECM-like molecules like collagen type IV, fibronectin or plasminogen/plasmin. Cholesterol depletion of HeLa cells had contrary effects on the adherence and cell invasion of *M. gallisepticum*, calling us to investigate the role of lipid rafts on the host cell surface in these mechanisms.

## Competing interests

The authors declare that they have no competing interests.

## Authors’ contributions

MPS and RR have designed the project and helped analyzing and interpreting the results. UF and KSG carried out the experimental work, UF performed the statistical analysis. UF and MPS wrote the manuscript which all authors have read and finally approved.
